# Puerperal Eclampsia*Read at meeting, Austin District Medical Society (Seventh Councilor’s), Austin, Texas, September, 1904.

**Published:** 1905-10

**Authors:** T. B. Taylor

**Affiliations:** Elgin, Texas


For Texas Medical Journal.

### Puerperal Eclampsia.*

BY T. B. TAYLOR, M. D., ELGIN, TEXAS.

"Puerperal Eclampsia," adopting Jewett's definition, is an acute morbid condition occurring during pregnancy, labor, or the puerperal state characterized by a series of tonic and clonic convulsions, affecting first, the voluntary and then the involuntary muscles, and accompanied by complete loss of consciousness and ending in coma or deep sleep.

It is more frequent during later months of pregnancy, less so, during labor, and in our personal experience, rarely occurs for the first time during the puerperium, or after the birth of the child. The ratio of its occurrence, as given by various authorities, ranges from 1 to 250, to 1 to 500, of all pregnancies, but certainly, our personal experience shows a far greater percentage of eclampsias than the figures given above. Those pregnant for the first time are far more apt to be attacked than are those who have borne children before, though neither class are in any sense exempt.

---

*Read at meeting, Austin District Medical Society (Seventh Councilor's), Austin, Texas, September, 1904.

### SYMPTOMS.

The symptoms must be divided into those of the pre-eclamptic state, and of the attack itself, which later may be further subdivided into (1), invasion (2), tonic and clonic convulsions, (3), coma, or deep sleep.

Of the first class of symptoms (those of the pre-eclamptic state) and by far the most important—since an ounce of prevention has always been said to be worth a pound of cure, for by proper attention to these we may never know of the attack itself, we have persistent headache, confined for the most part to the frontal regions, loss of appetite, tongue furred and coated, arterial tension high, bowels constipated, scanty urine almost always containing albumen, face and lower extremities oedemetous. Given the above symptoms and few of us would fail to recognize at once, that our patient was suffering from a toxemic condition, which, if not speedily relieved, would end in an eclamptic seizure. And right here I wish to stress the importance of a closer oversight of the obstetrical cases intrusted to our care. No woman expecting to become a mother, should ever be allowed to pass the seventh and eighth months of pregnancy without the direct personal oversight of her physician and a chemical analysis of her urine from two to four times, and yet, how few are the opportunities in which we have a chance to do these things, and, shall I say, "how slow we are to adopt them," even when opportunities offer.

Of the attack itself as indicated above, we have (1), invasion, in which the eyes stare, the lids twitch convulsively, the pupils, which at first may be firmly contracted, later dilate widely, the mouth is drawn to one side, the eyeballs now roll up, and then (2), there sets in tonic and clonic convulsions of the head and neck, gradually extending over the entire body, even to the opisthotonic curve, arms are extended and rigid, hands closed and thumbs drawn into the palms, the respiratory muscles are soon involved, and respiration may cease altogether, thus completing a horrid picture that is most trying upon family and friends and physician himself however long he may have practiced, or how often he may have met with such cases before. But I digress, after a few minutes in this condition, the patient passes rapidly into the stage (3) of coma, which lasts usually about thirty minutes, exceptionally, only one attack may occur.

### ETIOLOGY.

Of the etiology of puerperal eclampsia one standard author says, "the last word has by no means been spoken," but this much is sure, that eclampsia does not always depend upon albuminuria and kidney change, and that albuminuria does not always accompany the convulsions, and yet he states, that the kidneys are involved in two-thirds of all the cases and that 84 per cent of the urine contains albumen. Few patients in the pregnant condition escape from diminished excretion due no doubt to the increased demand made upon the maternal organism. Few of us there be, who have not seen many of the mental and nervous peculiarities of the pregnant woman disappear when once her excretory organs were aroused to a proper activity. While we may not be able to point out the precise toxin causing these phenomean, may it not be the products of the patient's own tissue which furnishes the poisons that cause them?

### PATHOLOGY.

From what has already been said, it naturally follows that the pathology is very obscure.

### DIAGNOSIS.

The diagnosis is comparatively easy, though a little carelessness might lead to a most serious error.

There are four conditions to which the pregnant woman is liable, and which may be mistaken for eclampsia, viz., epilepsy, hysteria, apoplexy, and meningitis, but a little care on the part of the physician will easily enable him to make the distinction.

### PROGNOSIS.

We all recognize the fact that puerperal eclampsia is a most serious affection hence, the prognosis is grave, at present, the maternal mortality is given at 30 per cent and of the child 50 per cent. Any pregnant woman suffering from a toxemic albuminuria, and the daily quantity of whose urine is steadily diminishing, is in great danger of an attack, as the albumen increases and the daily quantity of water passed diminishes, the danger becomes more imminent. Bourchard and Davis, of Philadelphia, have shown that the amount of urea voided is a better guide in prognosis than albumen, having found the toxic symptoms to diminish in proportion as the amount of urea voided increases. The earlier the attack occurs in pregnancy the graver the prognosis.

The prognosis is favorable according to one standard author, in which we heartily concur:

1. When the attacks are infrequent and mild.
2. When the child dies.
3. When the patient is conscious in the intervals.
4. When there is a small amount of albumen.
5. When there is a fall of the temperature.
6. When attacks occur late in labor, or after birth of a child.

The reverse of these conditions would, of course, render the prognosis unfavorable.

### TREATMENT.

From what has already been shown, it naturally follows that the treatment would resolve itself into prophylactic and curative.

Under the first head, prophylactic, the first indication is to reduce the amount of nitrogenous foods to a minimum, and this can be best done by an exclusive milk diet, which really we consider the sheet anchor at this stage—as improvement takes place, fish or white meats may be added. The second indication, to limit the production and absorption of toxic materials in the intestines and tissues of the body, and assist in their elimination by improving the action of the bowels, skin, liver and kidneys and lungs as well. We advise all of the exercise in the open air consistent with safety, an abundant supply of pure water, drink it freely, not because our patient wants it, but because she needs and must have it. For the bowels colocynth or something along that line, followed by a saline in the morning. For the liver there is nothing better than an occasional dose at night of the mild chloride of mercury. For the skin, warm and hot baths, hot packs or the hot air baths according to urgency of case. For the kidneys any good diuretic, buchu, stoneroot, acetate potash, taken in 4 to 6 ounces of water, or large doses of nitroglycerine will bring about increased diuresis. This line of treatment persisted in, will almost always relieve the toxemia of pregnancy, and thereby prevent an eclamptic seizure.

### CURATIVE.

In eclampsia we face a desperate condition, one requiring prompt and decisive action. To control the convulsions, there are four medicinal agents that stand out prominently, viz., chloroform, verat.

viride, chloral and morphine hypodermically. For the immediate control of the convulsions chloroform occupies first place, but second to chloroform only, is verat. viride; it reduces the pulse rate and it is said a convulsion is practically unknown with a pulse at or below 60. It reduces temperature, causes prompt diaphoresis and diuresis, so that it not only aids in controlling the convulsions, but in eliminating the unknown poison causing them as well. Ten to twenty minims of the Fl. Ex. P. D. & Co., or S. & D., should be given hypodermically every half hour until decided effects are shown, and then, just enough to maintain those effects. Twenty to forty grains of chloral may be used per rectum, but we believe a better plan would be to introduce a soft rectal tube into bowels as far as possible, and then throw in one to three gallons of warm normal salt solution.

One other remedy we have thus far not mentioned, venesection, much in vogue at one time, and under proper conditions and a suitable patient, very much in favor with a large per cent of physicians, even now. Prof. Hobart A. Hare is authority for the statement, that if verat. be used and intravenous transfusion of normal salt solution the indications for bleeding are more satisfactorily fulfilled than where blood is drawn from the veins.

All these failing, brings us of course to the rapid emptying of the uterus, and here I believe, we should make haste slowly, unless the conditions are entirely favorable. If the cervix be tightly shut, I don't believe we should interfere, but rather, if we rescue our patient from the toxemia, the pregnancy may be allowed to go on.

On the 1st of August last, I was called to see Mrs. N., a stout, robust primipara in her eighth month of pregnancy. She presented all the premonitory, and as it afterwards proved, pre-eclamptic symptoms herein described, face and lower extremities very oedematous, bowels constipated, tongue heavily coated, severe frontal headache, urine very scant and heavily loaded with albumen, skin very dry. Ordered warm bath and good rubbing with coarse towel, prescribing mild Chlor. Mer. Ext. Coloc. Co. Sacch. Lactis aa grs., 1. every hour till six doses should be taken, to be followed with large saline, 1 ounce, sulphate of magnesia, in hot water. This was at 10 a. m. At 4 p. m. I was hurriedly called to find Mrs. N. having convulsions, having warned her husband at my previous visit that they were likely to occur. I gave patient one-half grain morphine hypodermically and administered chloroform by inhalation. Not having a reliable preparation of verat. in my case, I returned to my office, about three blocks, to procure same, which I had some trouble in doing, causing delay. Upon my return I found Mrs. N. had had two very severe convulsions during my absence, the last just passing off. Unconscious she lay moaning, presenting a very undesirable picture. I gave her at once 20 drops of P. D. & Cos. fluid extract veratrum and repeated the dose in 40 minutes. Pulse dropped to 56, convulsions ceased; after that time I gave her 5 drops pr. orem every two to four hours; at 3 a. m. she was delivered of a large still born babe, and made an uneventful recovery.

